# Seneca Valley Virus 3C^**pro**^ Cleaves Heterogeneous Nuclear Ribonucleoprotein K to Facilitate Viral Replication

**DOI:** 10.3389/fmicb.2022.945443

**Published:** 2022-07-06

**Authors:** Jiangwei Song, Rong Quan, Dan Wang, Jue Liu

**Affiliations:** ^1^Beijing Key Laboratory for Prevention and Control of Infectious Diseases in Livestock and Poultry, Institute of Animal Husbandry and Veterinary Medicine, Beijing Academy of Agriculture and Forestry Sciences, Beijing, China; ^2^College of Veterinary Medicine, Yangzhou University, Yangzhou, China; ^3^Jiangsu Co-innovation Center for Prevention and Control of Important Animal Infectious Diseases and Zoonoses, Yangzhou University, Yangzhou, China

**Keywords:** seneca valley virus, heterogeneous nuclear ribonucleoprotein K, viral replication, host protein regulation, viral 3C protease

## Abstract

Seneca Valley virus (SVV) has emerged as an important pathogen that is associated with idiopathic vesicular infection in pigs, causing a potential threat to the global swine industry. The heterogeneous nuclear ribonucleoprotein K (hnRNP K) that shuttles between the nucleus and cytoplasm plays an important role in viral infection. In this study, we observed that infection with SVV induced cleavage, degradation, and cytoplasmic redistribution of hnRNP K in cultured cells, which was dependent on the activity of viral 3C^pro^ protease. Also, the 3C^pro^ induced degradation of hnRNP K via the caspase pathway. Further studies demonstrated that SVV 3C^pro^ cleaved hnRNP K at residue Q364, and the expression of the cleavage fragment hnRNP K (aa.365–464) facilitates viral replication, which is similar to full-length hnRNP K, whereas hnRNP K (aa.1–364) inhibits viral replication. Additionally, hnRNP K interacts with the viral 5′ untranslated region (UTR), and small interfering RNA (siRNA)-mediated knockdown of hnRNP K results in significant inhibition of SVV replication. Overall, our results demonstrated that the hnRNP K positively regulates SVV replication in a protease activity-dependent fashion in which the cleaved C-terminal contributes crucially to the upregulation of SVV replication. This finding of the role of hnRNP K in promoting SVV propagation provides a novel antiviral strategy to utilize hnRNP K as a potential target for therapy.

## Introduction

Seneca Valley virus (SVV) was initially isolated as a contaminant during adenovirus cultivation in the United States in 2002 (Hales et al., 2008). SVV mainly affects pigs and is associated with idiopathic vesicular disease in pigs. SVV infection has been frequently reported in the swine industry worldwide for the past 10 years and has led to huge economic losses (Pasma et al., 2008). In contrast to being a potential threat to the swine industry, SVV is a deliverable oncolytic agent used in the treatment of neuroendocrine cancers and invasive retinoblastoma (Reddy et al., 2007; Wadhwa et al., 2007).

Seneca Valley virus belongs to the genus *Senecavirus* of the family *Picornaviridae* and is a non-enveloped, single-stranded positive-sense RNA virus (Hales et al., 2008; Venkataraman et al., 2008). The genome is ~7.2 kb in length and comprises one open reading frame (ORF) that encodes a polyprotein that is cleaved into 12 proteins by viral-encoded 3C protease (3C^pro^) and cellular proteases (Hales et al., 2008). 3C^pro^ plays an important role in regulating the host's innate immunity (Qian et al., 2017; Xue et al., 2018; Wen et al., 2019), apoptosis (Fernandes et al., 2019; Liu et al., 2019), stress granules (Wen et al., 2020), autophagy (Wen et al., 2021b), and pyroptosis (Wen et al., 2021a). SVV 3C^pro^ targets mitochondrial antiviral signaling (MAVS), toll/interleukin 1 (IL-1) receptor domain-containing adaptor inducing IFN-(TRIF), and tumor necrosis factor receptor-associated factor (TRAF) family member-associated NF-κB activator (TANK) for cleavage to suppress host type I interferon production (Qian et al., 2017). Moreover, the retinoic acid-inducible gene I (RIG-I) was degraded by SVV 3C^pro^, which results in the inhibition of type I interferon production (Xue et al., 2018; Wen et al., 2019). The critical cellular proteins were cleaved and degraded by SVV 3C^pro^ to promote viral replication, like poly (A)-binding protein cytoplasmic 1 (PABPC1) (Xue et al., 2020), selective autophagy receptor SQSTM1/p62 (sequestosome 1) (Song et al., 2021a; Wen et al., 2021b), and gasdermin D (Wen et al., 2021a). PABPC1 and SQSTM1/p62 lost their antiviral activities after being cleaved by 3C^pro^ (Xue et al., 2020; Wen et al., 2021b). These cleavage sites preferentially localized in glutamine–glycine (Q-G) or glutamic acid–glutamine (E-Q) pairs in proteins, and the cleavage was dependent on the protease activity of 3C^pro^. Gasdermin D was targeted by SVV 3C^pro^ for cleavage, and both gasdermin D and cleaved gasdermin D (aa.1-277) possess bactericidal activities *in vivo* (Wen et al., 2021a). Additionally, SVV 3C^pro^ mediates apoptosis in the host cells and cleaves nuclear factor-kappa B (NF-κB)-p65 and poly (adenosine diphosphate-ribose) polymerase (PARP) (Fernandes et al., 2019). The formation of stress granules (SG) could be blocked by SVV 3C^pro^ by disrupting the eIF4GI–G3BP1 interaction (Wen et al., 2020). However, other cellular proteins regulated by 3C^pro^ that may also participate in SVV replication remain unclear.

The family of heterogeneous nuclear ribonucleoproteins (hnRNPs) has more than 20 members, named in sequence from hnRNP A to hnRNP U (Martinez-Contreras et al., 2007; Han et al., 2010); they participate in diverse cellular biological functions, such as chromatin remodeling, transcription, mRNA translation, splicing, and nuclear-cytoplasmic shuttling (Bomsztyk et al., 1997, 2004; Michael et al., 1997; Ostareck-Lederer and Ostareck, 2004). hnRNP K contains an RNA-binding domain, DNA-binding domain, and protein interaction domain (Ostareck-Lederer et al., 1998). hnRNP K participates in viral replication by interacting with the 5′ untranslated region (UTR) of enterovirus 71 (EV71), foot-and-mouth disease virus (FMDV), and hepatitis C virus (HCV) (Lin et al., 2008; Fan et al., 2014; Liu et al., 2020). Infections with EV71 and FMDV, members of the family of *Picornaviridae*, induce hnRNP K redistribution from the nucleus to the cytoplasm (Lin et al., 2008; Liu et al., 2020). In addition, hnRNP K relocalizes to the cytoplasm after infection with vesicular stomatitis virus (VSV), HCV, and Sindbis virus (Burnham et al., 2007; Pettit Kneller et al., 2009; Fan et al., 2014). Knockdown of hnRNP K significantly affects the release of infectious herpes simplex virus-1 virions into the extracellular environment (Schmidt et al., 2010). Several viral proteins interact with hnRNP K, including Chikungunya virus (CHIKV) nsP2, Sindbis virus nonstructural proteins, HCV core protein, African swine fever virus (ASFV) p30, and bovine ephemeral fever virus (BEFV) nonstructural protein α3 (Hsieh et al., 1998; Burnham et al., 2007; Hernaez et al., 2008; Bourai et al., 2012; Jiang et al., 2020). FMDV and EV71 infection induce the cleavage and degradation of hnRNP K to regulate viral replication (Jagdeo et al., 2018; Liu et al., 2020). However, whether cellular hnRNP K contributes to the efficient replication of SVV in cultured cells remains unknown.

In this study, we found that SVV infection causes cleavage, degradation, and redistribution of hnRNP K in the cultured cells. Further studies have shown that 3C^pro^ mediates these actions through its protease activity. Downregulation of hnRNP K expression significantly inhibited SVV replication, whereas hnRNP K overexpression promoted viral propagation. Overall, these findings indicate that the cellular hnRNP K is involved in the efficient replication of SVV through its cleavage and redistribution induced by SVV 3C protease activity, which provides novel insights into the replication mechanisms of SVV regulated by host proteins.

## Results

### SVV Infection Cleaves hnRNP K and Induces hnRNP K Degradation

**Figure 1:**
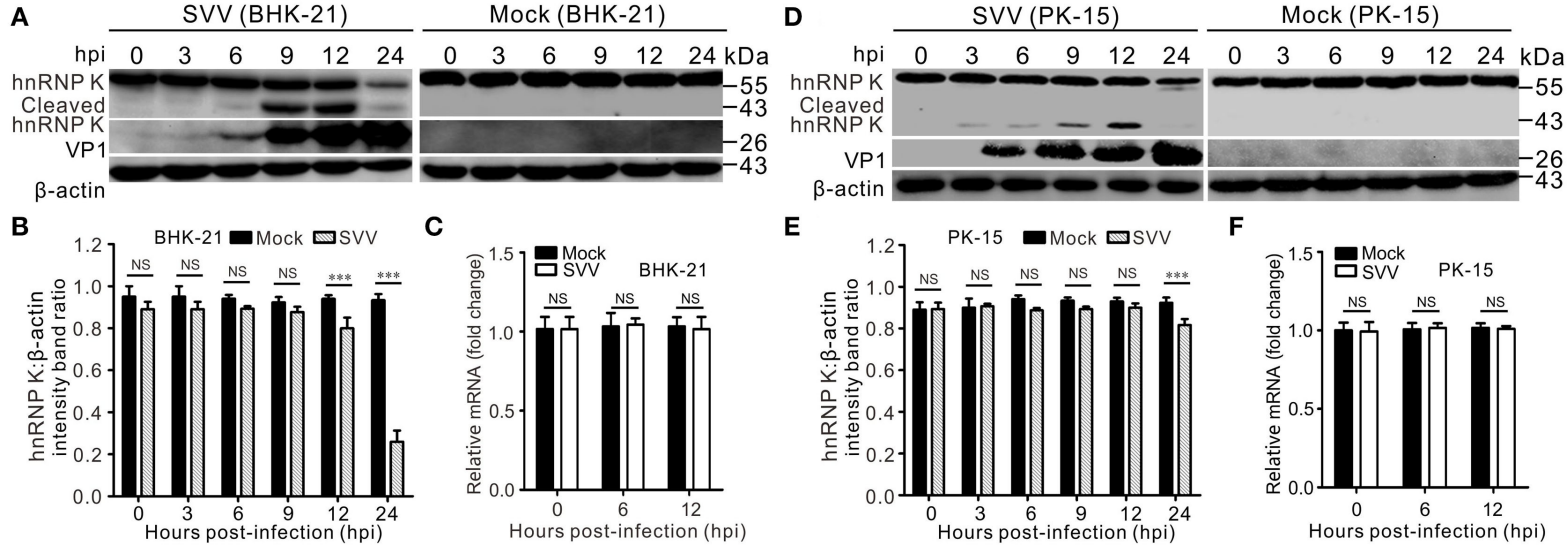
SVV infection cleaves hnRNP K and induces hnRNP K degradation. **(A,D)** SVV-infected (MOI = 5) or mock-infected BHK-21 or PK-15 cells were extracted at 0, 3, 6, 9, 12, and 24 hpi, and protein samples were subjected to Western blotting with antibodies against hnRNP K, VP1, and β-actin. **(B,E)** Graphs of quantification analyzed with Image J, the level of hnRNP K band ratios normalized to β-actin. Statistical analysis using GraphPad Prism. Results are displayed as the mean ± standard deviation (SD) of three independent tests (***P < 0.001; NS, not significant). **(C,F)** SVV-infected (MOI = 5) or mock-infected BHK-21 cells were harvested at 0, 6, and 12 hpi and the mRNA levels of hnRNP K were quantified by qRT-PCR. Statistical analysis using GraphPad Prism. Results are displayed as the mean ± SD of three independent experiments (**P < 0.01; NS, not significant).

Numerous hnRNPs are cleaved and degraded during viral infections (Jagdeo et al., 2015; Li et al., 2019; Liu et al., 2020). In a previous study, we found that SVV infection decreased the abundance of hnRNP A1 (Song et al., 2021b). To test whether SVV infection causes cleavage and degradation of hnRNP K, BHK-21 and PK-15 cells were infected with SVV, and hnRNP K degradation at various time points was assessed using western blotting. The results showed that hnRNP K levels gradually decreased during SVV infection (Figures 1A–E). We observed distinctly cleaved hnRNP K bands at 9 h post-infection (hpi), and the levels of cleaved hnRNP K increased until 12 hpi (Figures 1A,D). Similar to full-length hnRNP K, the levels of cleaved hnRNP K were significantly reduced at 24 hpi (Figures 1A,D). However, hnRNP K mRNA levels were not significantly reduced (Figures 1C,F). This indicated that the reduction in hnRNP K was not attributed to a decrease in its mRNA accumulations. These results suggested that hnRNP K was cleaved and degraded in SVV-infected cells.

### SVV Infection Induces Cytoplasmic Relocalization of hnRNP K

**Figure 2:**
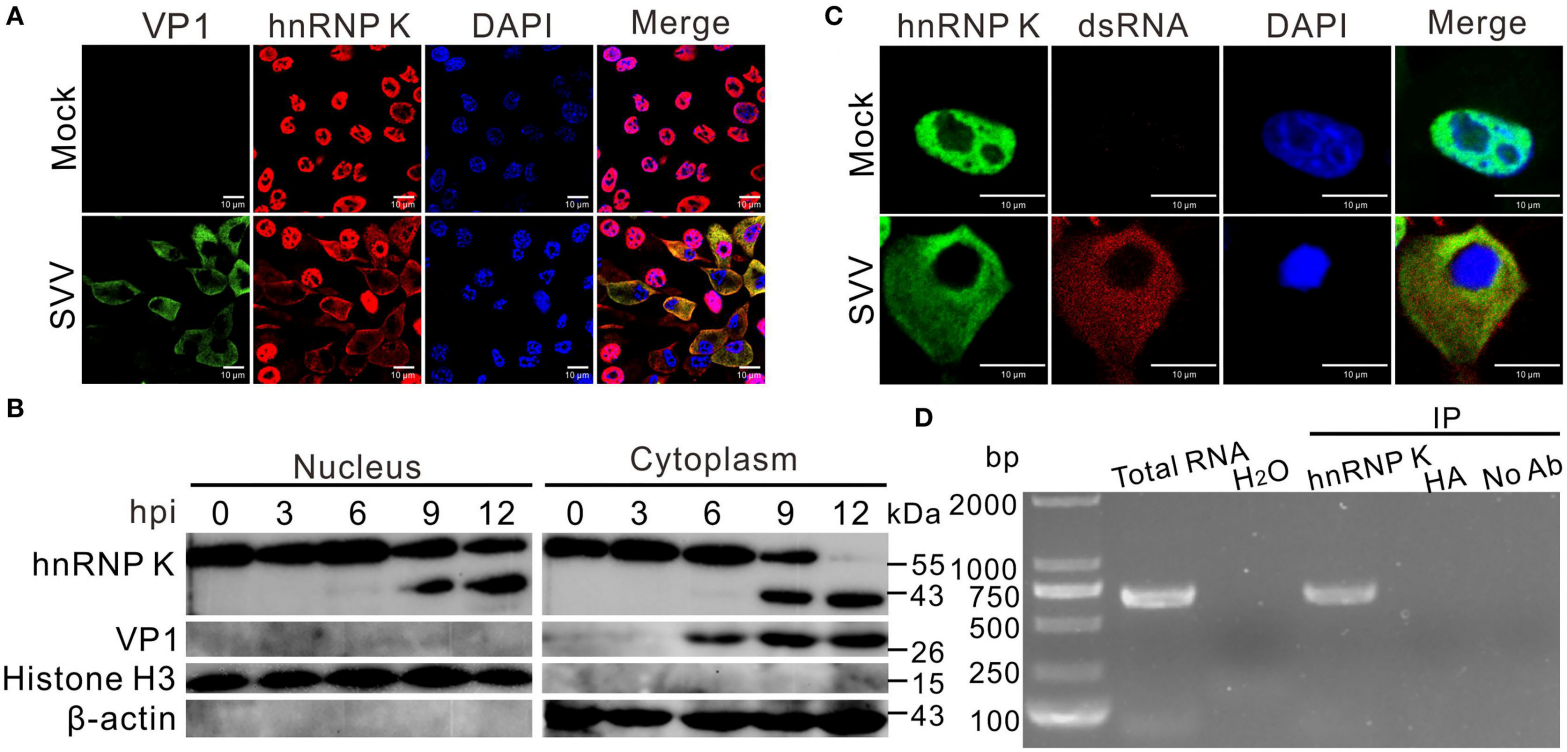
SVV infection induces relocalization of hnRNP K. **(A)** BHK-21 cells were infected with SVV (MOI = 5), at 0 and 12 hpi, cells were stained with antibodies against hnRNP K (red), VP1 monoclonal antibody (green), and DAPI (blue), captured under confocal microscopy. **(B)** SVV-infected (MOI = 5). BHK-21 cells were collected at 0, 3, 6, 9, and 12 hpi, cytoplasmic and nuclear fractions were extracted and subjected to Western blotting with antibodies against hnRNP K, VP1, histone H3, and β-actin. **(C)** BHK-21 cells were infected with SVV (MOI = 5), at 0 and 12 hpi, cells were stained with antibodies against hnRNP K (green), dsRNA monoclonal antibody (red), and DAPI (blue), captured under confocal microscopy. **(D)** SVV 5′UTR RNA was pulled down with hnRNP K from SVV-infected (MOI = 5) cell lysate. BHK-21 cells were infected with SVV for 12 h, the harvested cell lysates were immunoprecipitated with rabbit anti-hnRNP K, rabbit anti-HA, and without antibodies, then subjected to RT-PCR. SVV-infected cells and H_2_O used as a positive control and negative control for RT-PCR using SVV 5′UTR specific primers, respectively.

To verify the response of hnRNP K to SVV infection, an immunofluorescence assay was performed to examine the subcellular localization of hnRNP K. We found that hnRNP K was not within the nuclei, but was present in the cytoplasm after SVV infection (Figure 2A). The ratio of nucleocytoplasmic relocalization of hnRNP K dramatically increased after SVV infection. Consistent with cytoplasmic redistribution and cleavage, the cytoplasmic and nuclear fractions of western blot analysis demonstrated that cytoplasmic hnRNP K was primarily the cleaved form at 12 hpi (Figure 2B). The redistributed hnRNP K in the cytoplasm colocalized with viral dsRNA, indicating a possible interaction of hnRNP K with the viral genome (Figure 2C). To determine whether hnRNP K binds to the SVV 5′ UTR, an RNA–protein co-immunoprecipitation assay was performed; in the assay, a specific DNA band was amplified in the immunoprecipitation complexes with an anti-hnRNP K antibody, but not anti-hemagglutinin (HA) antibody or without antibody (Figure 2D). Collectively, these data demonstrated that hnRNP K is capable of interacting with the 5′ UTR in SVV-infected cells, and that infection with SVV induces cytoplasmic translocation and retention of hnRNP K.

### SVV 3C^pro^ Cleaves and Degrades hnRNP K, and Retains hnRNP K in the Cytoplasm

**Figure 3:**
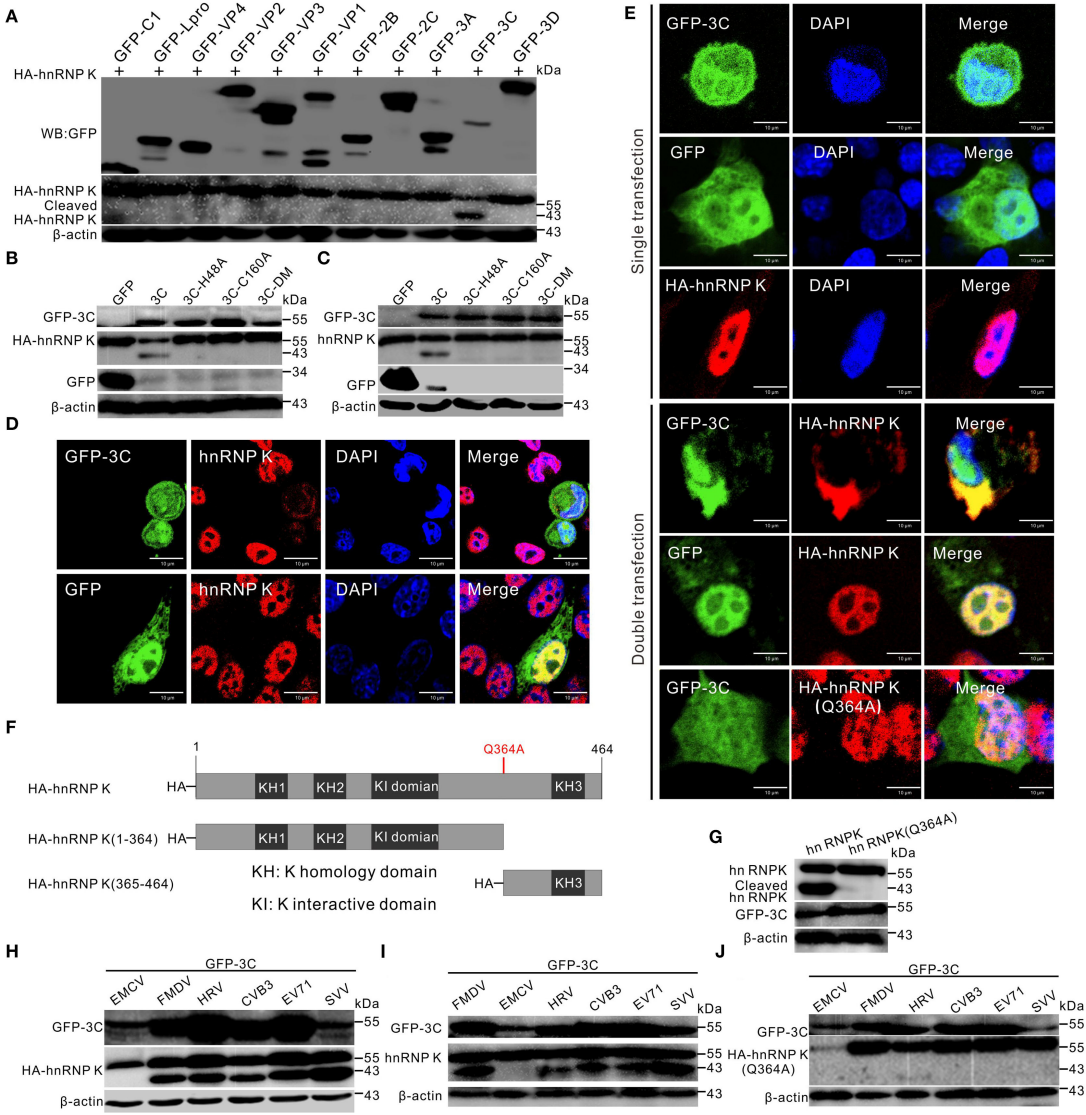
SVV 3C^pro^ cleaves hnRNP K. **(A)** BHK-21 cells were cotransfected with HA-hnRNP K and GFP-viral gene plasmids, the cells were collected by Western blotting analysis at 24 h post-transfection (hpt). **(B)** BHK-21 cells in six-well plates were cotransfected with HA-hnRNP K and GFP empty vector, GFP-3C, GFP-3C-H48A, GFP-3C-C160A, or GFP-3C-DM. The cell lysates were subjected to Western blotting at 24 hpt. **(C)** BHK-21 cells in six-well plates were transfected with GFP empty vector, GFP-3C, GFP-3C-H48A, GFP-3C-C160A, or GFP-3C-DM. The cell lysates were subjected to Western blotting at 24 hpt. **(D)** BHK-21 cells were transfected with GFP-3C (green) and GFP vector (green) and stained with antibodies against hnRNP K (red) and DAPI (blue), captured under confocal microscopy. **(E)** BHK-21 cells were single- or double- transfected with GFP-3C (green), GFP vector (green), HA-hnRNP K, or HA-hnRNP K (Q364A), stained with antibodies against HA (red) and DAPI (blue), captured under confocal microscopy. **(F)** Domain organization and engineering of truncation constructs of HA-hnRNP K. KH, K homology domain; KI, interactive domain. **(G)** BHK-21 cells were cotransfected with HA-hnRNP K and HA-hnRNP K (Q364A) with GFP-3C. The cells were collected by Western blotting at 24 hpt. **(H)** HA-hnRNP K was cotransfected with GFP-3C of EMCV, FMDV, HRV, CVB3, EV71, and SVV plasmids into BHK-21 cells, the cells were collected by Western blotting at 24 hpt. **(I)** BHK-21 cells were transfected with GFP-3C of EMCV, FMDV, HRV, CVB3, EV71, and SVV plasmids into BHK-21 cells, the cells were collected by Western blotting at 24 hpt. **(J)** HA-hnRNP K (Q364A) was cotransfected with GFP-3C of EMCV, FMDV, HRV, CVB3, EV71, and SVV plasmids into BHK-21 cells, the cells were collected by Western blotting at 24 hpt.

Seneca Valley virus infection can degrade numerous cellular proteins, and 3C^pro^ is one of the key viral proteins responsible for the degradation (Qian et al., 2017; Wen et al., 2019, 2021b). We conducted investigations to identify the viral proteins responsible for cleavage and cytoplasmic relocalization of hnRNP K. Initially, individual viral proteins were screened for their ability to cleave hnRNP K. We found that SVV 3C^pro^ could cleave hnRNP K and induce hnRNP K degradation *in vitro* (Figure 3A). To determine whether the protease activity of 3C^pro^ was responsible for degradation, BHK-21 cells were co-transfected with HA-hnRNP K and GFP-3C-H48A, GFP-3C-C160A, or GFP-3C-DM (H48A-C160A), as described previously (Song et al., 2021b). The results demonstrated that catalytic histidine (his48) and cysteine (cys160) residues in 3C^pro^ mediated the degradation of hnRNP K, and on losing their catalytic activities, they lost their ability to cleave and degrade hnRNP K *in vitro* and *in vivo* (Figures 3B,C). These findings demonstrated that 3C^pro^-mediated cleavage of hnRNP K is dependent on the protease activity of 3C^pro^. Endogenous degradation was examined using immunofluorescence assay by transfection of GFP-3C and incubated with antibodies against hnRNP K. Transfection of GFP-3C decreased immunofluorescence signals (red) of hnRNP K, whereas, transfection of GFP vector did not affect endogenous hnRNP K (Figure 3D). These results indicate that 3C^pro^ can induce hnRNP K degradation *in vivo*. In co-transfected BHK-21 cells, GFP-3C caused cytoplasmic retention of hnRNP K and was perfectly colocalized with it in the cytoplasm (Figure 3E). In contrast, hnRNP K that was predominantly localized in the nucleus did not respond to GFP-3C (Figure 3E). These results suggested that 3C^pro^ is responsible for hnRNP K redistribution. SVV 3C^pro^ preferentially targets glutamine–glycine (Q-G) or glutamic acid–glutamine (E-Q) pairs for cleavage (Qian et al., 2017; Wen et al., 2021b). According to informatics analysis, one Q residue (Q364) was replaced with A (Q364A) within hnRNP K (Figure 3F). The Q364A mutation in hnRNP K was resistant to cleavage and translocation after co-transfection with GFP-3C, confirming that Q364 is recognized by SVV 3C^pro^ for hnRNP K cleavage and cytoplasmic redistribution (Figures 3E,G). We also assessed other members of the picornavirus family and found that the 3C^pro^ of FMDV, human rhinovirus (HRV), coxsackievirus B3 (CVB3), and EV71 play the same role as that of SVV in cleaving HA-hnRNP K and endogenous hnRNP K (Figures 3H,I), while 3C^pro^ of EMCV cannot cleave hnRNP K (Figures 3H,I). Moreover, the Q364 residue of hnRNP K was recognized by 3C^pro^ of FMDV, HRV, CVB3, and EV71 for cleavage (Figure 3J). Together, these results revealed that SVV 3C^pro^ cleaves and degrades hnRNP K depending on its protease activity.

### SVV 3C^pro^ Degrades hnRNP K Through the Caspase Pathway

**Figure 4:**
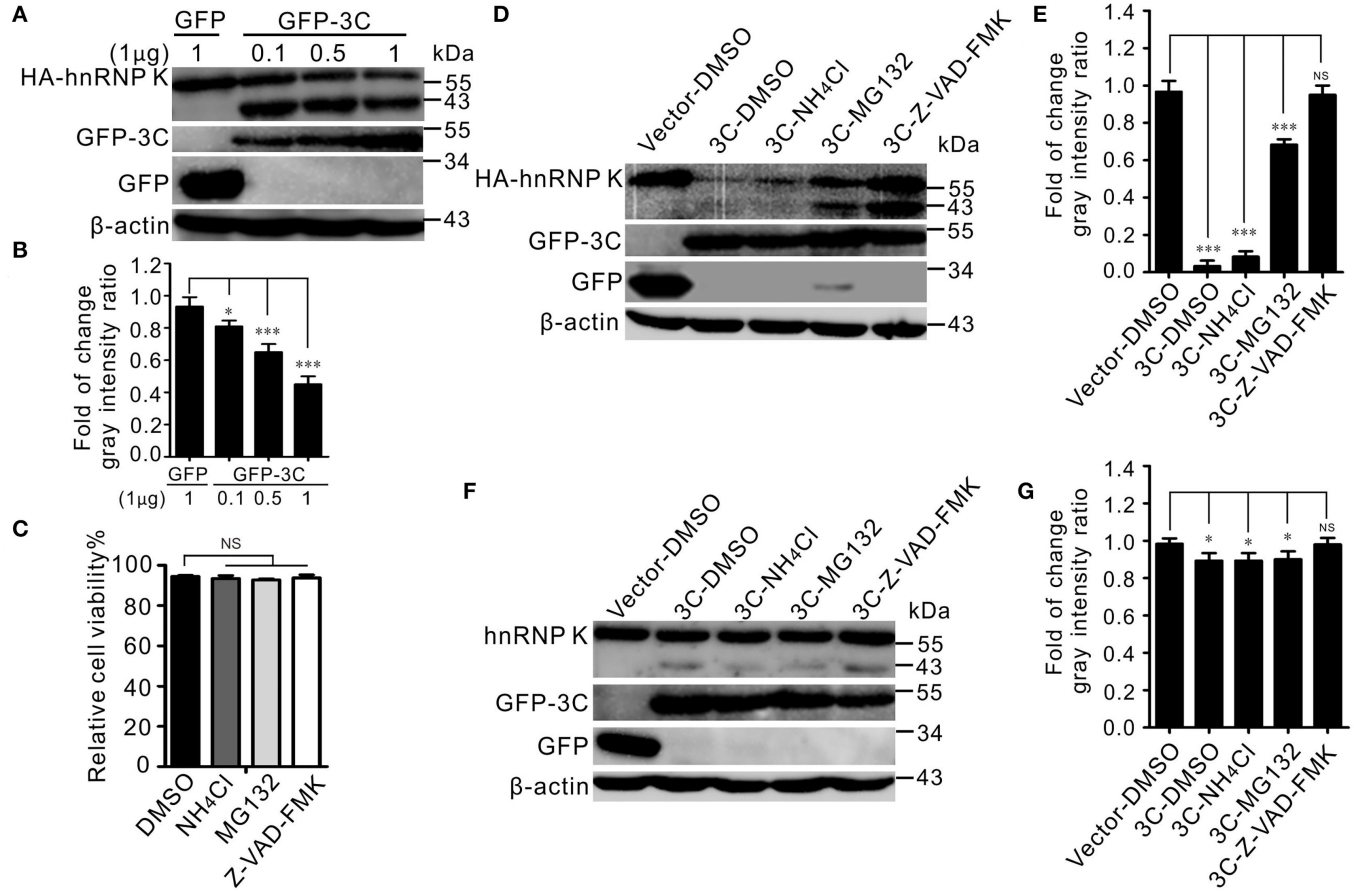
SVV 3C^pro^ meditated hnRNP K degradation via its enzyme activity, through the caspase pathway. **(A)** BHK-21 cells in six-well plates were cotransfected with 1 μg HA-hnRNP K and 0.1, 0.5, and 1 μg GFP-3C or empty vector, respectively. The cell lysates were subjected to Western blotting at 24 hpt. **(B)** The ratios of HA-hnRNP K normalized against β-actin of three independent experiments of **(A)** (*P < 0.05; ***P < 0.001). Graphs of quantification analyzed with Image J. Data are represented by mean ± SD. **(C)** The viability of BHK-21 cells was analyzed using CCK-8 assay after treatment with chemical reagents. Error bars indicate the mean ± SD of three independent experiments (NS, not significant). **(D)** BHK-21 cells in six-well plates were cotransfected with HA-hnRNP K and GFP empty vector, or GFP-3C. At 10 hpt, cells were treated with DMSO, Z-VAD-FMK (50 μM), MG132 (10 μM), and NH_4_Cl (10 mM). The cell lysates were subjected to Western blotting at 24 hpt. **(E)** The ratios of HA-hnRNP K normalized against β-actin of three independent experiments of **(D)** (*P < 0.05; ***P < 0.001). Graphs of quantification analyzed with Image J. Data are represented by mean ± SD. **(F)** BHK-21 cells in six-well plates were transfected GFP empty vector, or GFP-3C. At 10 hpt, cells were treated with DMSO, Z-VAD-FMK (50 μM), MG132 (10 μM), and NH_4_Cl (10 mM). The cell lysates were subjected to Western blotting at 24 hpt. **(G)** The ratios of hnRNP K normalized against β-actin of three independent experiments of **(F)** (*P < 0.05; ***P < 0.001). Graphs of quantification analyzed with Image J. Data are represented by mean ± SD.

Previous studies have shown that SVV 3C^pro^ degrades cellular proteins depending on its protease activity (Qian et al., 2017; Wen et al., 2019; Song et al., 2021b). Different doses of GFP-3C were co-transfected with HA-hnRNP K, and the results indicated that 3C^pro^ degraded hnRNP K in a dose-dependent manner (Figures 4A,B). The degradation pathway was explored with the proteasome inhibitor MG132, caspase inhibitor Z-VAD-FMK, and lysosome inhibitor NH_4_Cl. The cytotoxicity of the inhibitors in BHK-21 cells was tested using the CCK-8 assay, and it was found that the cell viability was not affected (Figure 4C). The degradation was significantly alleviated in the presence of the caspase inhibitor Z-VAD-FMK *in vitro* (Figures 4D,E). In contrast, treatment with the lysosome inhibitor NH_4_Cl and proteasome inhibitor MG132 had no evident influence on degradation *in vitro* (Figures 4D,E). The endogenous degradation pathway was in accordance with the *in vitro* degradation results (Figures 4F,G). These data showed that the 3C^pro^-mediated degradation of hnRNP K occurs via the caspase pathway.

### hnRNP K Is Critical for SVV Replication

**Figure 5:**
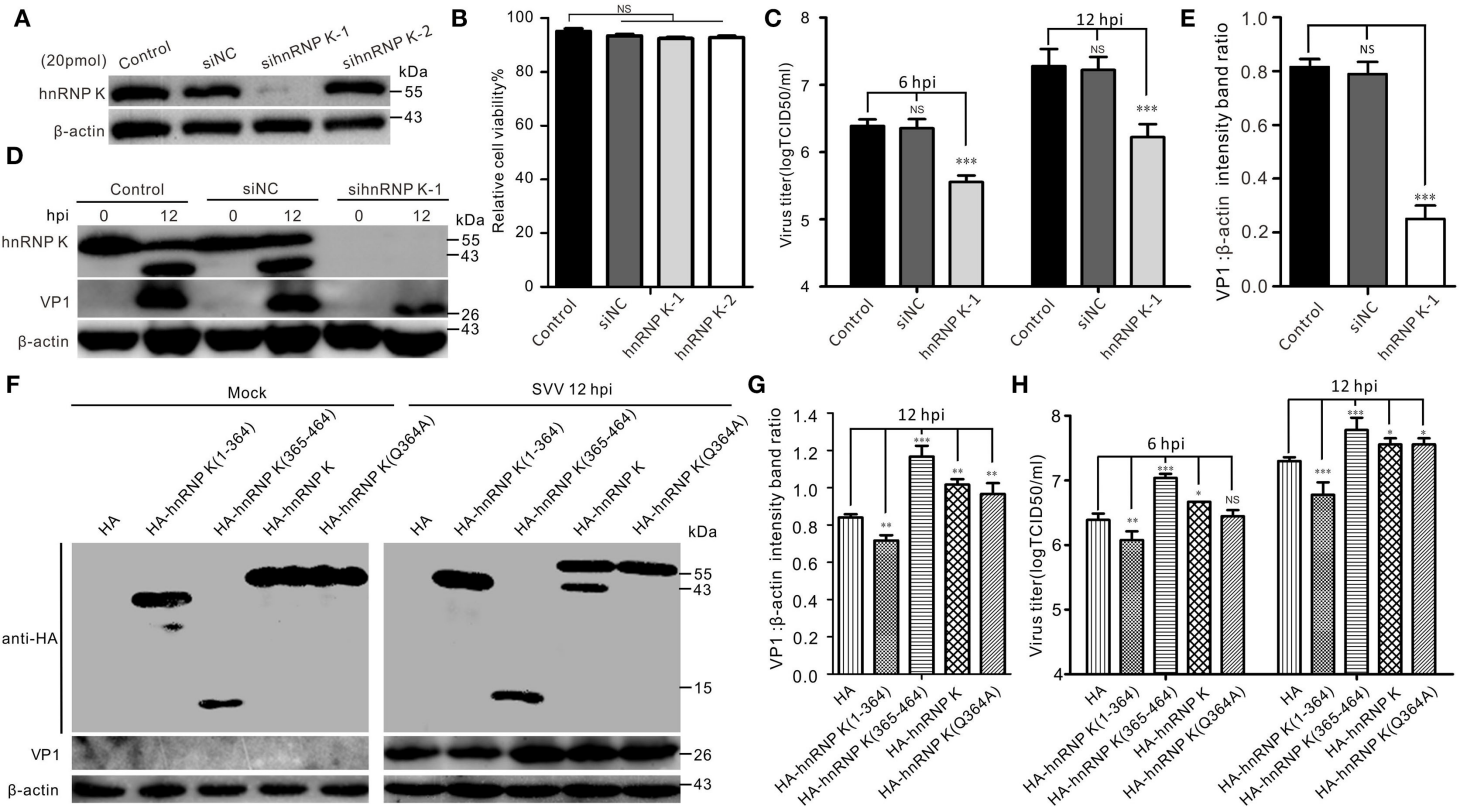
The expression of hnRNP K is essential for SVV replication. **(A)** BHK-21 cells in six-well plates were mock-transfected or transfected with 20 pmol of hnRNP K-siRNA and negative control (NC)-siRNA for 48 h. The expression of hnRNP K was assessed by Western blotting. **(B)** The viability of si-RNA transfected BHK-21 cells was analyzed using CCK-8 assay. Error bars indicate the mean ± SD of three independent experiments (NS, not significant). **(C)** BHK-21 cells transfected with siRNA for 48 h were infected with SVV (MOI = 1), and the virus productions were assessed by TCID_50_. Error bars indicate the mean ± SD of three independent experiments (NS, not significant). **(D)** BHK-21 cells transfected with siRNA for 48 h were infected with SVV (MOI = 5), and the expression of VP1 was assessed by Western blotting. **(E)** The ratios of VP1 normalized against β-actin of three independent experiments of **(C)** (*P < 0.05; ***P < 0.001). Graphs of quantification analyzed with Image J. Data are represented by mean ± standard deviation (SD). **(F)** BHK-21 cells were transfected HA-hnRNP K (1–364), HA-hnRNP K (365–464), HA-hnRNP K, HA-hnRNP K (Q364A), and empty HA vector. At 24 hpt, the cells were infected with SVV (MOI = 5), and the cell lysates were subjected to Western blotting at 12 hpi. **(G)** The ratios of VP1 normalized against β-actin of three independent experiments of **(F)** (*P < 0.05; ***P < 0.001). Graphs of quantification analyzed with Image J. Data are represented by mean ± SD. **(H)** BHK-21 cells were transfected with HA-hnRNP K (1–364), HA-hnRNP K (365–464), HA-hnRNP K, HA-hnRNP K (Q364A), and empty HA vector. At 24 hpt, the cells were infected with SVV (MOI = 1), and the supernatants were titrated by TCID_50_ assay at 12 hpi.

Given the importance of hnRNP K in SVV infection, we examined hnRNP K expression using an RNA interference (RNAi) assay with two different small interfering RNAs (siRNAs) targeting hnRNP K. The results showed that only one siRNA efficiently knocked down hnRNP K (Figure 5A). Importantly, siRNA transfection had no effect on cell viability (Figure 5B). Compared with siNC (negative control, NC) and mock-transfected BHK-21 cells, downregulation of hnRNP K significantly decreased the virus titer and VP1 protein expression (Figures 5C–E). Finally, we comprehensively assessed the effects of cleaved hnRNP K (1–364), cleaved hnRNP K (365–464), hnRNP K (Q364A), and full-length hnRNP K on viral replication. Consistent with the cleavage of HA-hnRNP K *in vitro* (Figure 3A), transfected HA-hnRNP K was observed to be cleaved after SVV infection, whereas HA-hnRNP K (Q364A) was not (Figure 5F). Contrary to RNAi-mediated knockdown, overexpression of hnRNP K enhanced viral replication (Figures 5F–H). Similar to the full-length hnRNP K, hnRNP K (365–464) and hnRNP K (Q364A) had the same influence on SVV replication (Figures 5F–H). However, hnRNP K (1–364) inhibits SVV replication (Figures 5F–H). Collectively, these results suggested that hnRNP K expression facilitates SVV replication.

## Discussion

The family of hnRNPs is a type of nucleocytoplasmic shuttling RNA-binding protein primarily located in the nucleus. To date, more than 20 hnRNPs have been identified, named hnRNP A to hnRNP U (Martinez-Contreras et al., 2007; Han et al., 2010), which play important roles in the life cycle of various viruses (Hsieh et al., 1998; Brunner et al., 2005; Burnham et al., 2007; Hernaez et al., 2008; Lin et al., 2008, 2009; Fan et al., 2014; Brunetti et al., 2015; Jagdeo et al., 2015, 2018; Li et al., 2018, 2019; Chiu et al., 2019; Liu et al., 2020). The present study suggested that the proteolytic cleavage of hnRNP K mediated by 3C^pro^ of picornavirus is a common strategy and that SVV hijacks hnRNP K to facilitate viral replication.

Our results showed that SVV infection induced hnRNP K cleavage and caused hnRNP K degradation (Figures 1A,D). Almost all hnRNP K in the nucleus relocalized to the cytoplasm during SVV infection at 12 hpi, while hnRNP K remained in the nucleus of cells with mock infection (Figure 2A). Cytoplasmic translocation of the hnRNP family of proteins upon infection with picornaviruses may share common replication strategies. hnRNP M is redistributed in the cytoplasm after infection with poliovirus and coxsackievirus (Lin et al., 2008, 2009; Jagdeo et al., 2015). hnRNP K relocalizes to the cytoplasm and interacts with the FMDV internal ribosomal entry site (IRES) to inhibit IRES-mediated translation (Liu et al., 2020). Dengue virus type 2 (DENV-2) and Junin virus (JUNV) infection induces subcellular redistribution of hnRNP K, whereas the expression of hnRNP K is unaffected (Brunetti et al., 2015). Therefore, it would be interesting to understand the cytoplasmic factors that drive the translocation of hnRNP K to the cytoplasm. hnRNP K translocated to the cytoplasm colocalized with the viral dsRNA (Figure 2C); a similar finding was observed after infection with FMDV (Liu et al., 2020). A cytoplasmic nuclear fractionation assay was used to measure the cellular distribution of hnRNP K during a time-course infection of SVV. At 6 hpi, only trace amounts of cleaved hnRNP K were detected in the nucleus, and its expression was significantly increased at 9 hpi in the nucleus and cytoplasm (Figure 2B). At 12 hpi, the majority of hnRNP K in the cytoplasm was cleaved (Figure 2B). RNA immunoprecipitation assays revealed that hnRNP K interacted with the 5′ untranslated region (5′ UTR) of coxsackievirus A16, FMDV, and EV71 (Lin et al., 2008; Li et al., 2016; Liu et al., 2020), and we also found that hnRNP K interacted with the 5′ UTR of SVV (Figure 2D). The results indicated that hnRNP K is a key cellular factor involved in the replication of picornaviruses through its interaction with the viral 5′ UTR. Relocalization of hnRNP K in the cytoplasm results in interaction with the 5′ UTR of EV71 to regulate viral replication (Lin et al., 2008), and hnRNP A1, which is distributed in the cytoplasm, promotes IRES-dependent translation of EV71 (Lin et al., 2009). hnRNP K binds to IRES to negatively regulate translation and replication of FMDV, and is antagonized by 3C^pro^. Further studies involving the interaction of hnRNP K with the SVV 5′ UTR are needed to understand the precise function of hnRNP K in SVV replication.

Screening of viral proteins revealed that 3C^pro^ is responsible for the cleavage of hnRNP K (Figure 3A). We constructed three mutant types of 3C^pro^ that lost their protease activity, as described in our previous study (Song et al., 2021b). Abrogation of the catalytic sites of 3C^pro^ abolished its proteolytic activity and degradation (Figures 4B,C), suggesting that the conserved catalytic residues of 3C^pro^ were responsible for 3C^pro^-mediated cleavage and degradation. Previous studies have shown that the hnRNP family is degraded and cleaved by catalytic residues, such as EV71 3C^pro^ targeting hnRNP A1 (Li et al., 2019), poliovirus 3C^pro^ targeting hnRNP M (Jagdeo et al., 2015), and CVB3 3C^pro^ targeting hnRNP K (Jagdeo et al., 2018). These results demonstrated that picornavirus 3C^pro^ targets the hnRNP family for cleavage and degradation universally. The red fluorescence nearly disappeared in GFP-3C transfected cells of immunofluorescence assay showing that SVV 3C^pro^ induced endogenous hnRNP K degradation (Figure 3D). In addition, 3C^pro^ induced the translocation of hnRNP K, resembling SVV-infected cells (Figures 2A, 3E). 3C^pro^ cleaved hnRNP K at the Q364 residue (Figures 3F,G), which was in accordance with the FMDV 3C^pro^-recognized hnRNP K cleavage site (Liu et al., 2020). These results add to the growing evidence that SVV 3C^pro^ recognizes glutamine–glycine (Q-G) or glutamic acid–glutamine (E-Q) sites for cleavage (Blom et al., 1996). 3C^pro^ targets Q-G to cleave host proteins such as SQSTM1/p62 (Wen et al., 2021b). 3C^pro^ from several picornaviruses were used to evaluate the cleavage of hnRNP K. The results indicated that hnRNP K could not be cleaved by EMCV 3C^pro^, and the 3C^pro^ of FMDV, HRV, CVB3, and EV71 induced the same cleaved bands as SVV (Figures 3H,I), which is in accordance with previous studies (Jagdeo et al., 2018; Liu et al., 2020). Picornavirus 3C^pro^ cleaves other members of the hnRNP family, including hnRNP A1 (Li et al., 2019), hnRNP D [AU-rich binding factor 1, AUF1; (Rozovics et al., 2012)], hnRNP E2 [poly (rC)-binding protein 2, PCBP2; (Chase and Semler, 2014)], hnRNP I [polypyrimidine tract-binding protein, PTB; (Back et al., 2002)], and hnRNP M (Jagdeo et al., 2015).

Seneca Valley virus 3C^pro^ degrades and cleaves numerous cellular factors depending on its catalytic residues (Qian et al., 2017; Xue et al., 2018, 2020; Li et al., 2019; Wen et al., 2019). We demonstrated here that 3C^pro^ downregulated hnRNP K in a dose-dependent manner. The expression of hnRNP K was significantly restored in the presence of Z-VAD-FMK (Figures 4D,E), indicating that degradation depended on the caspase pathway. Treatment with the proteasome inhibitor MG-132 and the lysosome inhibitor NH_4_Cl did not alleviate degradation. Degradation of hnRNP K during BEFV infection is mediated by a caspase 3-dependent pathway (Jiang et al., 2020). Our previous study determined that SVV 3C^pro^ degrades hnRNP A1 via the proteasome pathway (Song et al., 2021b). This indicates that the degradation pathway of hnRNPs is variable within the hnRNP family. We speculate that hnRNP K and hnRNP A1 might have different molecular structures and functions. They are distinct from other hnRNP proteins. hnRNP K is bound to numerous cellular proteins via its K interactive (KI) domain (Bomsztyk et al., 2004). hnRNP A1 utilizes two RNA recognition motifs for RNA binding in its N-terminus, while hnRNP K utilizes three K homology (KH) domains, two in the N-terminus and one in the C-terminus (Geuens et al., 2016). Although hnRNP K and hnRNP A1 belong to the family of hnRNPs, they played different roles in the life cycle of EV71. For instance, hnRNP A1 stimulates EV71 IRES activity and influences the synthesis of viral proteins, while hnRNP K is necessary for the efficient synthesis of viral RNA (Shih et al., 2011). hnRNP K and hnRNP A1 can interact with the EV71 5′UTR for EV71 propagation (Lin et al., 2008, 2009). hnRNP K interacts with cloverleaf structure and stem-loop IV within IRES (Lin et al., 2008), but hnRNP A1 interacts with stem-loops II and VI in IRES of EV71, and not with the cloverleaf structure (Lin et al., 2009). EV71 3C^pro^-mediated cleavage of hnRNP A1 might turn off the translation of some specific cellular mRNAs unless it is substituted with hnRNP A2 (Lin et al., 2009). Both hnRNP K and hnRNP A1 interact with the 5′UTR of Coxsackievirus A16, which is important for translational activity (Li et al., 2016). These results demonstrated that hnRNPs members play different roles in regulating picornavirus replication.

Silencing hnRNP K expression significantly impaired the replication of dengue virus and Junín virus (Brunetti et al., 2015). For picornavirus, siRNA-mediated knockdown of hnRNP K and hnRNP M inhibited EV71 and poliovirus replication, respectively (Lin et al., 2008; Jagdeo et al., 2015). In contrast, hnRNP K downregulation promotes FMDV replication (Liu et al., 2020). This indicates that hnRNP K has different effects on the replication of picornaviruses depending on the type of virus. RNAi-mediated knockdown and overexpression indicated that hnRNP K plays a crucial role in the regulation of SVV replication (Figure 5). One of the cleavage products, hnRNP K (1–364) negatively regulates replication (Figures 5F–H). The two proteolytic products of hnRNP K have opposing roles in regulating viral replication. A previous study showed that the full-length and cleaved N-terminal hnRNP K interacted with FMDV IRES to reduce viral replication but the cleaved C-terminal hnRNP K did not, suggesting that negative regulation of hnRNP K in FMDV replication might be due to fine balance of these two cleaved terminals of hnRNP K established during FMDV infection (Liu et al., 2020). In contrast to the role of hnRNP K in decreasing FMDV replication, we find that hnRNP A upregulates SVV replication and speculate that positive regulation of the full-length and cleaved C-terminal hnRNP K may be partially attenuated by negative regulation of the cleaved N-terminal hnRNP K, thereby maintaining the balance to facilitate SVV propagation. As shown in Figure 3F, the N-terminal hnRNP K (1-364) consists of KH1, KH2, and KI domains, but the C-terminal hnRNP K (365-464) only includes the KH3 domain. The KH domain is an RNA-binding domain that interacts with single-stranded RNA (Shih et al., 2011). What makes hnRNP K different from the other hnRNPs is that it binds tenaciously to poly (C) sequences. However, it will be essential to investigate the precisely-regulated mechanism of the full-length hnRNP K and two cleaved hnRNP K fragments during SVV infection in the future.

In conclusion, we found that SVV infection downregulated the expression of hnRNP; it cleaved and induced the translocation of hnRNP K. Degradation and cleavage were mediated by the protease activity of 3C^pro^ through the caspase pathway. Overall, our results provided important insights into the role of the host factor hnRNP K in SVV replication and potential antiviral targets for the prevention of SVV infection.

## Materials and Methods

### Cells, Viruses, Antibodies, and Chemical Reagents

BHK-21 cells and PK-15 cells were cultured at 37°C in Dulbecco's modified Eagle's medium (DMEM; Invitrogen, CA, USA) supplemented with 10% fetal bovine serum (FBS) (Invitrogen). The SVV strain CHhb17 and mouse monoclonal antibody against SVV VP1 were preserved in our laboratory (Hou et al., 2019). Antibodies for β-actin (mouse monoclonal; A1978) and dsRNA antibody (mouse monoclonal; MABE1134) were purchased from Sigma-Aldrich (St. Louis, MO, USA). Antibodies for hnRNP K (rabbit polyclonal; 11426-1-AP) were purchased from Proteintech (Wuhan, China). Antibodies for histone H3 antibody (rabbit polyclonal; ab183902) and GFP (mouse monoclonal; ab127417) were purchased from Abcam (Cambridge, MA, USA). Goat anti-rabbit immunoglobulin (IgG)-horseradish peroxidase (ab205719) and goat anti-mouse IgG-horseradish peroxidase (ab205718) secondary antibodies were purchased from Abcam. Goat anti-rabbit immunoglobulin (IgG)-Alexa-568, goat anti-mouse IgG-Alexa-568, goat anti-mouse IgG-Alexa-488, and goat anti-rabbit IgG-Alexa-488 secondary antibodies were purchased from Molecular Probes (Invitrogen). Proteasome inhibitor MG132 and caspase inhibitor Z-VAD-FMK were purchased from Selleck Chemicals (Shanghai, China).

### Plasmid Construction

The hnRNP K gene was cloned and inserted into the pCMV-HA vector (Clontech, 631604) with ClonExpress One Step Cloning Kit (Vazyme, C112, China). GFP-infused SVV proteins were reported in our previous study (Song et al., 2021a,b, 2022). Mutagenesis of HA-hnRNP K to generate HA-hnRNP K (Q364A) using KOD DNA polymerase (TOYOBO, KFX-201), GFP-3C-H48A, GFP-3C-C160A, and GFP-3C-DM (H48A and C160A double mutants) were reported in our previous study (Song et al., 2021b). The 3C sequence of EMCV, FMDV, rhinovirus, CVB3, and EV71 were synthesized from Tsingke Biotechnology (Beijing, China) and recombined into the vector pEGFP-C1 that was used in our previous study (Song et al., 2022). The primers used for this study are listed in Table 1.

**Table 1:** Primers used in this study.

**Primers^**a**^**	**Sequence (5^**′**^-3^**′**^)^**b**^**	**Restriction site**
HA-hnRNP K-F	TGGCCATGGAGGCCCGAATTCGGATGGAGACCGAGCAGCCAGA	EcoRI
HA-hnRNP K-R	GATCCCCGCGGCCGCGGTACCTTAGAATCCTTCAACATCTGCATA	KpnI
HA-hnRNP K (365-464)-F	TGGCCATGGAGGCCCGAATTCGGATGGGTGGTTCTGGATATGATT	EcoRI
HA-hnRNP K (1-364)-R	GATCCCCGCGGCCGCGGTACCTCACTGTGGTTCATAAGCCATCTGC	KpnI
hnRNP K-Q364A-F	AGATGGCTTATGAACCAGCGGGTGGTTCTGGATATGATTA	
hnRNP K-Q364A-F	TAATCATATCCAGAACCACCCGCTGGTTCATAAGCCATCT	

a *F denotes forward primer; R denotes reverse primer*.

b *Restriction sites are underlined*.

### Western Blotting

Cells were harvested and lysed using lysis buffer (0.5% NP-40, 50 mM Tris, 0.5 mM EDTA, 150 mM NaCl) containing a protease inhibitor (1 mM phenylmethylsulfonyl fluoride, PMSF), and the lysates were incubated at 4°C with a shaker for 30 min. Proteins were fractionated by sodium dodecyl sulfate polyacrylamide gel electrophoresis (SDS-PAGE) followed by a wet transfer onto nitrocellulose (NC) membranes. Membranes were blocked with phosphate-buffered saline (PBS) containing 5% nonfat milk, then incubated with specific primary antibodies, and then the membranes were washed three times with PBS containing 0.2% Tween 20. Horseradish peroxidase (HRP)-conjugated secondary antibodies and chemiluminescence detection reagents (Thermo Fisher, 34096) were used to detect bound antibodies.

### Quantitative Real-Time PCR

Total RNAs of SVV-infected cells were extracted by using TRIzol reagent (Invitrogen). A total of 1 μg of total RNA was subjected to cDNA synthesis and qPCR was determined using SYBR qPCR Mix (Vazyme, China). β-Actin served as a gene for endogenous reference. The primer sequences for β-actin are as follows: forward primer 5′-TTCAACACCCCAGCCATGTACG-3′ and reverse primer 5′- CAGCCAGGTCCAGACGCAGGAT-3′ and for hnRNP K: forward primer 5′- TGCAGTTGCCATCACCCACTGCAA-3′ and reverse primer 5′- CCAATAATTCCTCCTGCCAGACT -3′.

### Immunofluorescence Assay (IFA)

BHK-21 cells grown on coverslips were infected with SVV or transfected with plasmids. At the indicated time, the culture medium was removed, cells were washed with PBS, and then fixed with 4% paraformaldehyde (PFA) for 8 min at room temperature (RT). After being washed with PBS, the cells were permeabilized with 0.1% Triton X-100 for 8 min (RT) and then blocked with 2% bovine serum albumin (BSA) in PBS for 30 min (RT). The samples were reacted with primary and secondary antibodies for 1 h sequentially (RT). The images were captured using a Nikon A1 confocal microscopy.

### Nuclear and Cytoplasmic Fractionation

SVV-infected BHK-21 cells were collected at different times postinfection, and nuclear and cytoplasmic fractionation were prepared using the nuclear and cytoplasmic extraction reagents (Thermo Fisher, 78833) according to the manufacturer's instructions. The fractions were analyzed using western blotting with antibodies against histone-H3 and β-actin, the markers for cytoplasmic and nuclear proteins, respectively.

### RNA-Protein Coimmunoprecipitation

BHK-21 cells were infected with SVV for 12 h and then lysed by lysis buffer (0.5% NP-40, 50 mM Tris, 0.5 mM EDTA, 150 mM NaCl) containing a protease inhibitor (1 mM PMSF). Samples were centrifugated at 14,000 rpm for 10 min at 4°C. Cell lysates were preincubated with protein A/G-agarose (Santa Cruz) at 4°C with a shaker for 30 min. The protein A or G beads were removed by centrifugation at 1,000 g at 4°C for 5 min. The supernatant was incubated with protein A/G-agarose and rabbit anti-hnRNP K, rabbit anti-HA, or control rabbit IgG antibody and incubated overnight at 4°C on a rocker. After washing with wash buffer, RNA was extracted from the beads–protein–RNA complexes with TRIzol reagent. RT-PCR was used to analyze the interaction with primers for the SVV 5′UTR (forward primer 5′-TTGAATGGGGGGCTGGGCCCTCAT-3′ and reverse primer 5′- ATTTGTATGTGCTACCTATAGAAC-3′).

### RNA Interference (RNAi)

Small interfering RNAs (siRNAs) targeting hamster hnRNP K were synthesized in GenePharma (Suzhou, China): si-hnRNP K-1 (sense, 5′-CCCUGCAGAAGAUAUGGAATT-3′; antisense, 5′-UUCCAUAUCUUCUGCAGGGTT-3′) and si-hnRNP K-2 (sense, 5′-GCUGACAGAGUCAUACUUATT-3′; antisense, 5′-UAAGUAUGACUCUGUCAGCTT-3′). The negative-control siRNA (sense, 5′-UUCUCCGAACGUGUCACGUTT-3′; antisense, 5′-ACGUGACACGUUCGGAGAATT-3′). hnRNP K siRNA was transfected using Lipofectamine RNAiMAX (Thermo Fisher, 13778150).

### Statistical Analysis

Statistical significance used in this work was evaluated using GraphPad Prism (version 5.0; La Jolla, CA, USA). All data are reported as mean ± standard deviation (SD) with a *p* < 0.05 set as statistical significance.

## Data Availability Statement

The raw data supporting the conclusions of this article will be made available by the authors, without undue reservation.

## Author Contributions

JL and JS conceived, designed the experiments, drafted the manuscript, and revised the manuscript. JS and DW performed the experiments. RQ and JS were responsible for the statistical analysis of the data. All authors reviewed the manuscript. All authors contributed to the article and approved the submitted version.

## Funding

This work was supported by the Innovation Capacity of Beijing Academy of Agriculture and Forestry Sciences (KJCX20220411), Foundation of Key Laboratory of Livestock Infectious Diseases in Northeast China (Shenyang Agricultural University), Ministry of Education (FKLID-2021-03), and the Priority Academic Program Development of Jiangsu Higher Education Institutions (PAPD).

## Conflict of Interest

The authors declare that the research was conducted in the absence of any commercial or financial relationships that could be construed as a potential conflict of interest.

## Publisher's Note

All claims expressed in this article are solely those of the authors and do not necessarily represent those of their affiliated organizations, or those of the publisher, the editors and the reviewers. Any product that may be evaluated in this article, or claim that may be made by its manufacturer, is not guaranteed or endorsed by the publisher.
